# The Three-Dimensional Evaluation of Radicular Cyst by Cone-Beam Computed Tomography

**DOI:** 10.7759/cureus.36162

**Published:** 2023-03-14

**Authors:** Arun Viggness, Sasti Priyaa, Ravikumar Pethagounder Thangavelu, Saramma Mathew Fenn, Karthik Rajaram Mohan

**Affiliations:** 1 Oral Medicine and Radiology, Vinayaka Mission's Sankarachariyar Dental College, Vinayaka Mission's Research Foundation (Deemed to be University), Salem, IND; 2 Oral Medicine, Vinayaka Mission's Sankarachariyar Dental College, Vinayaka Mission's Research Foundation (Deemed to be University), Salem, IND

**Keywords:** odontogenic cyst, root canal treatment, curettage, radicular cyst, cell rests of malassez

## Abstract

A radicular cyst is the most typical odontogenic cyst affecting the human jaws. A radicular cyst is frequently asymptomatic and is discovered accidentally during a radiological procedure. Radicular cyst most commonly occurs during the third and fourth decades of life. The patient affected by a radicular cyst usually gives a history of trauma, and they may even not be aware of the occurrence of the traumatic episode. Such a case of a Radicular cyst that occurred in a 22-year-old-young female who did not follow up further for root canal treatment was radiographically evaluated in three-dimensional view with the help of cone-beam computed tomography.

## Introduction

The radicular cyst is also known by other names, such as a periapical cyst or root-end cyst, as it occurs in the periapical region or the root end of the affected tooth. The radicular cyst is usually asymptomatic and is accidentally discovered by routine radiographic examination. Patients affected by radicular cyst usually complain of swelling in the vicinity of the affected tooth region by such cyst. Some patients may even complain of pain in the affected tooth region if the radicular cyst is infected. Radicular cyst occurs more commonly among males in the anterior maxilla region. The teeth commonly affected by radicular cyst are the ones that are more prone to trauma, including the maxillary central incisor, maxillary lateral incisor, mandibular central incisor, and mandibular lateral incisor. The patient usually gives a history of trauma; some even forget the history of the traumatic episode. Here we report a case of radicular cyst in a 22-year-old-female, who underwent an access opening root canal treatment procedure in a nearby private dental clinic four years back but has not followed further follow-up visits [1-3].

## Case presentation

**Figure 1:**
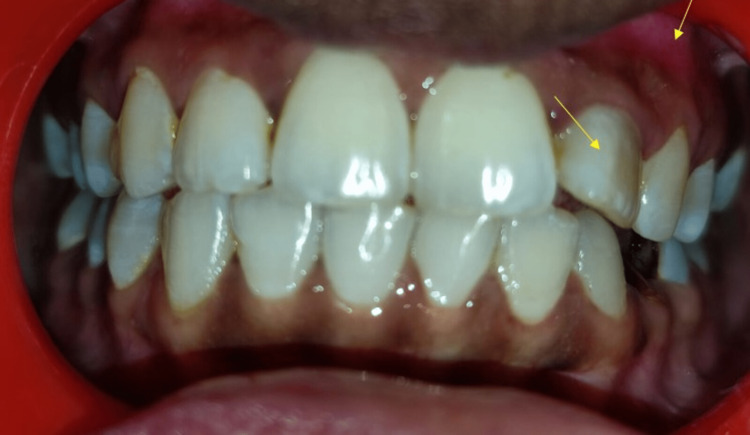
Intraoral examination revealed discoloured tooth 22 and obliteration of labial vestibule due to the presence of intraoral swelling in relation to 22 and slight labial tilt of 22

A 22-year-old-female reported a chief complaint of pain in the upper front tooth region for two weeks. History reveals that she underwent a root canal treatment in the upper front teeth region four years back but has not followed up further visits properly. A general examination revealed her vitals are stable. The extraoral examination did not reveal any facial asymmetry. Intraoral examination revealed discoloured tooth 22 and obliteration of the labial vestibule due to intraoral swelling concerning the 22 region. There is also a slight labial tilt of 22 compared to the adjacent maxillary teeth (Figure 1).

**Figure 2:**
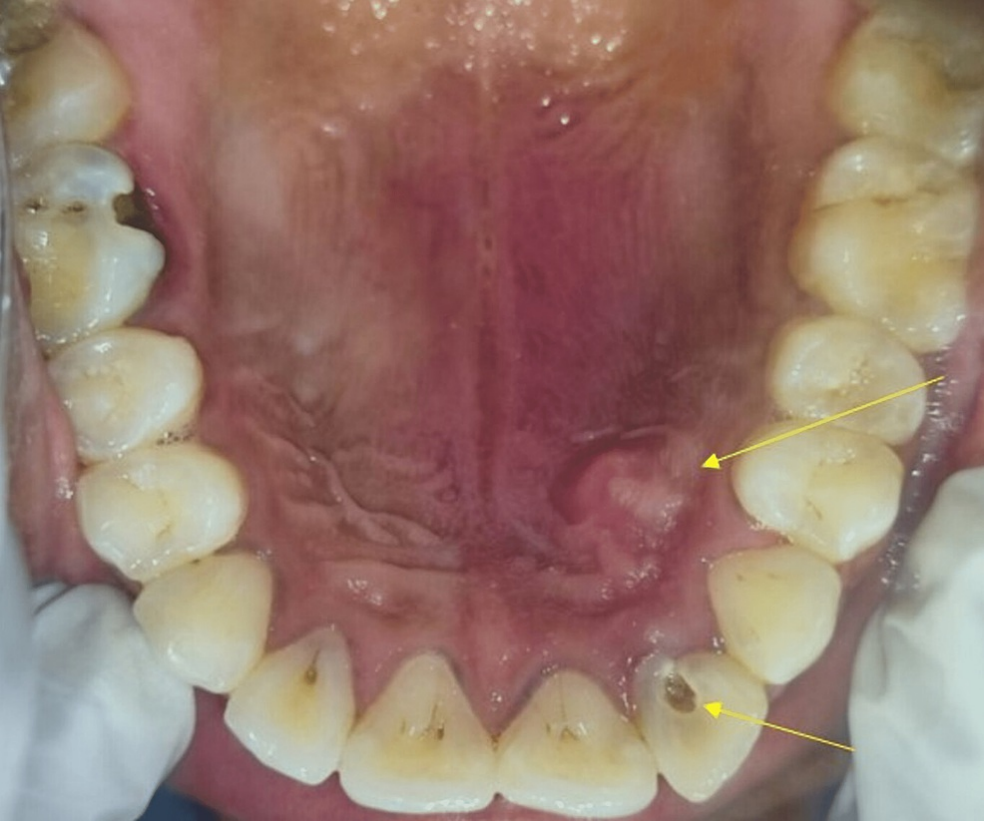
Intraoral examination revealed a localised intraoral swelling on the left anterolateral aspect of hard palate in relation to root canal attempted tooth 22

Further examination revealed a swelling localized on the hard palate on the palatal aspect of access opened teeth 22, measuring about 1.5 x 1 cm. The intraoral swelling extended anteriorly about 1 cm away from the marginal gingiva; medially, the swelling extends 1.5 cm away from the mid-palatal raphe region; posteriorly, the swelling extends 4.5 cm away from the posterior palatal seal area, laterally it extends 5 mm away from the marginal gingiva concerning 13, 14 tooth region. In addition, the intraoral swelling is firm, fluctuant, and tender on palpation (Figure 2).

**Figure 3:**
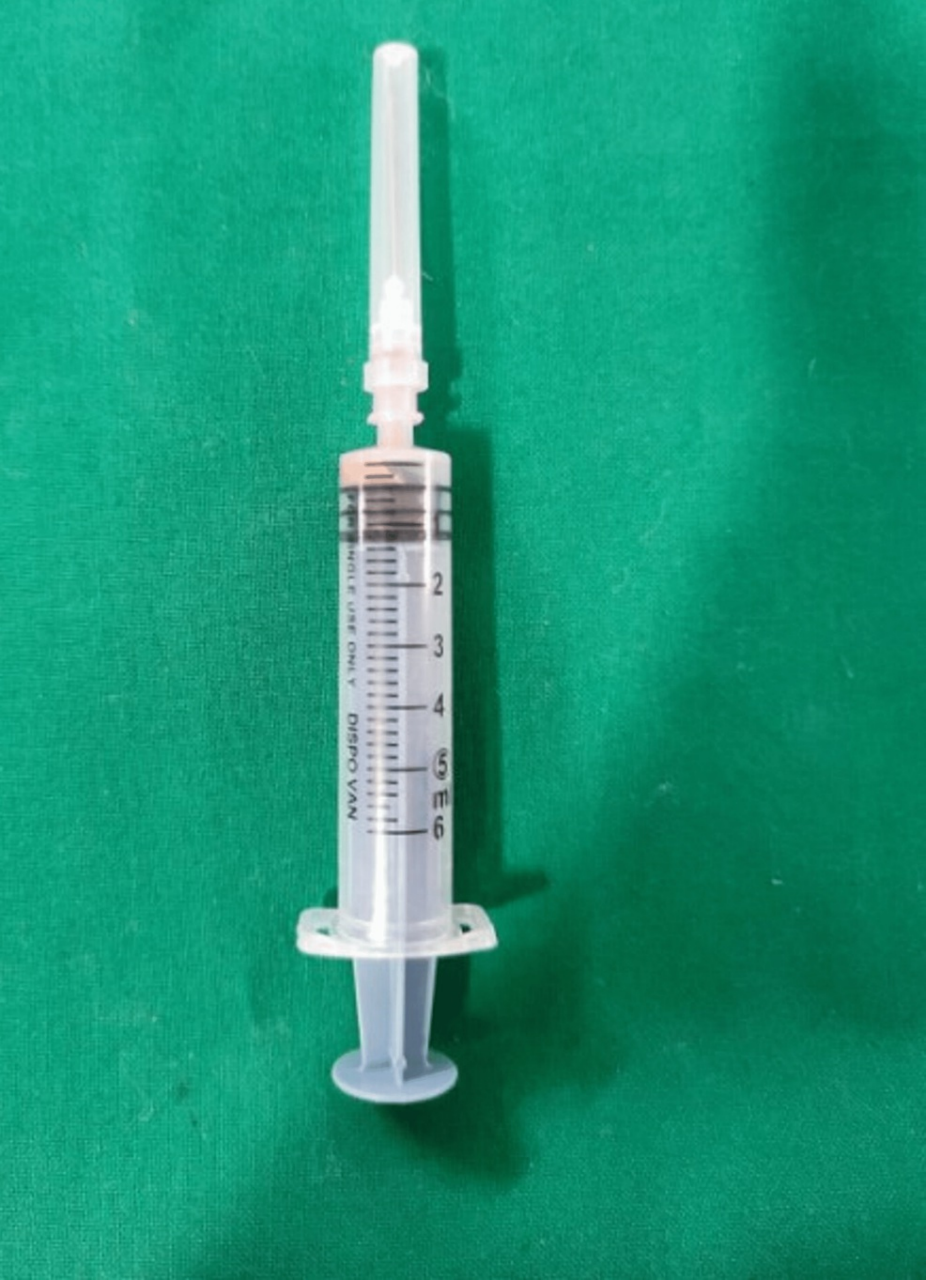
Aspiration with a 10-gauge wide bore needle revealed a blood -tinged straw-coloured fluid

On aspiration of the intraoral swelling revealed straw-coloured fluid mixed with blood, suggestive of an infected radicular cyst (Figure 3).

**Figure 4:**
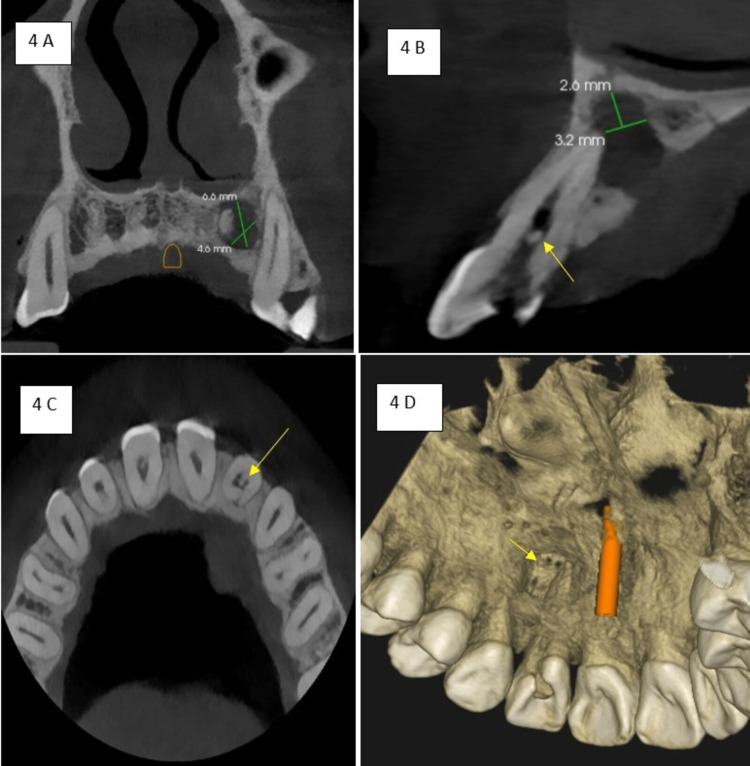
(A).Coronal revealed well-defined radiolucency measuring 4.6 x 6.6 mm. (B) Sagittal CBCT revealed well-defined radiolucency measuring about 3.2 mm x 2.6 mm with loss of lamina dura and palatal cortical plate perforation and pulp stone. (C) Axial section CBCT revealed a pulp stone in the coronal portion of pulp chamber in 22. (D) Three-dimensional reconstructed CBCT image revealed palatal cortical plate perforation in relation to 22.

The coronal section cone-beam computed tomography (CBCT) at a slice thickness of 75 micrometres at the level of the apical third of the root region of 22 revealed a well-defined radiolucency measuring about 4.6 mm x 6.6 mm surrounding the apical third root portion of 22. The sagittal section CBCT at a slice thickness of 1.1 mm revealed a well-defined radiolucency measuring about 3.2 x 2.6 mm concerning the apical region of the root of 22 with evident destruction of the palatal cortical plate and a discrete radiopacity measuring 1mm in diameter within the coronal portion of the pulp chamber, suggestive of a pulp stone. The axial section CBCT at 1.1 mm slice thickness at the level of the coronal portion of 22 also revealed a discrete radiopacity within the coronal portion of the pulp chamber in 22, suggestive of pulp stone. The Three -dimensional reconstructed CBCT image also revealed an evident palatal cortical plate destruction concerning 22 (Figures 4A-4D).

Periapical curettage was done in relation to the periapical region of 22 under infiltration with 2% lidocaine local anaesthesia. Postoperative medications cap. Amoxicillin (500 mg) t.d.s for a week and Tab. Ketorolac 10 mg b.i.d were prescribed for three days. The patient refused root canal treatment in 22.

**Figure 5:**
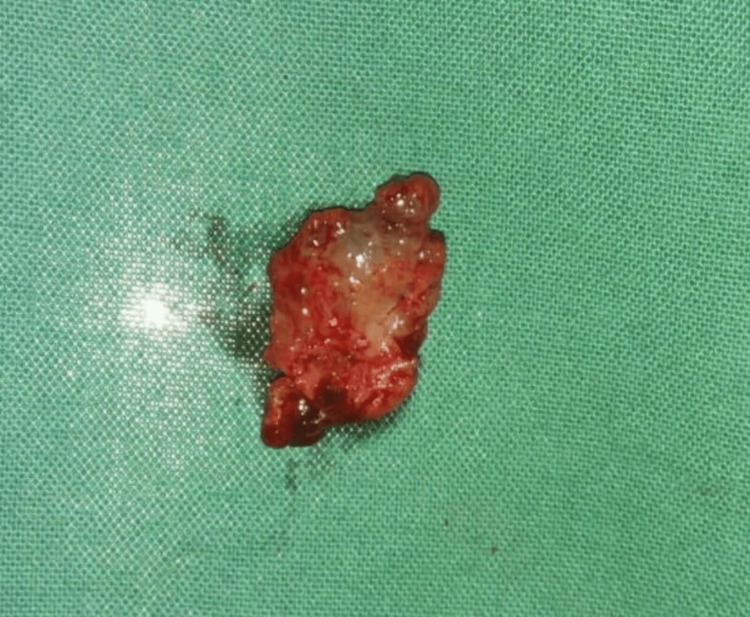
Excised specimen

**Figure 6:**
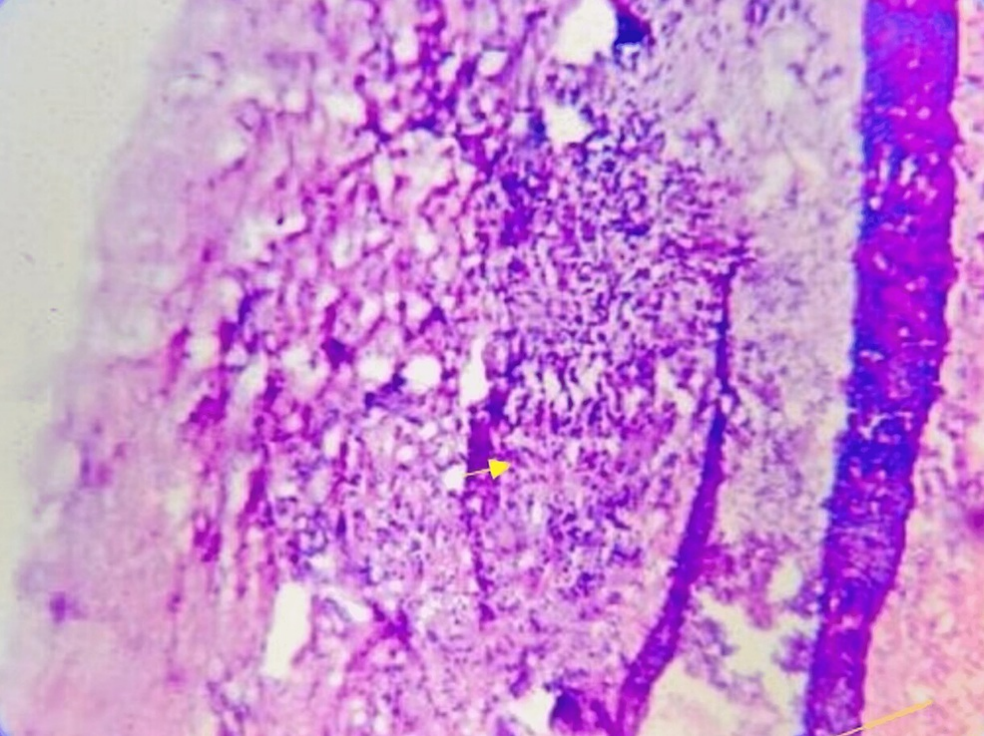
Histopathological photomicrograph (5x) revealed a cystic cavity containing an eosinophilic material infiltrated by numerous inflammatory cells such as neutrophils, lymphocytes, multinucleated giant cells, and cholesterol clefts

The excised specimen was sent for histopathological examination (Figure 5). Histopathological photomicrograph (10x) revealed a cystic lumen with an eosinophilic material containing numerous inflammatory cells like neutrophils, lymphocytes, multinucleated giant cells and cholesterol clefts (Figure 6).

The patient was relieved from tooth pain, and no swelling recurred on the hard palate in the 22 region.

## Discussion

The word “cyst,” which is derived from the Greek “Kystis,” means “sac or bladder.” A cyst is a diseased hollow pathological cavity bordered with epithelium that grows in a centrifugal, expansive manner [1]. The radicular cyst is the most common inflammatory odontogenic cyst in the human jaw. It is known by other names such as “Periapical cyst,” as it occurs in the periapical region of the affected tooth. “Root end cyst” occurs at the end of the tooth's root. The radicular cyst is more common among males in the anterior region of the maxilla or mandible, as it is the most common site for trauma. It is crucial to note that dental caries not only result in forming a radicular cyst, but trauma can also induce the formation of a radicular cyst. The radicular cyst usually causes swelling, which may not be prominent initially. Still, if the treatment is neglected, it can gradually cause cortical expansion, devitalize the adjacent teeth, and result in pathological migration of the teeth. The pathogenesis of radicular cyst is controversial. Although radicular cyst results from periapical infection from a deep carious tooth involving pulp, it also occurs in cases of teeth that had undergone a traumatic episode with an intact crown. The two theories that were postulated in the pathogenesis of radicular cyst include breakdown theory and abscess cavity theory, the former states that continuous growth of proliferating epithelial cell rests of Malassez occurs due to the crushing injury to the periodontal ligament fibers, due to trauma, which deprives the centrally located cells of acquiring their nutrition, resulting in cell death. As a result, liquefaction necrosis follows the centrally located cells, resulting in a radicular cyst formation. The latter states that an abscess-like cavity gets formed by the proliferating epithelial cell rests of Malassez, which arise from the remnant of periodontal ligament fibres and cover the underlying connective tissue. The tooth that underwent a severe impact trauma but is not fractured or dislocated has more chance of losing its pulp vitality than the fractured tooth [2-4].

The blood vessels of the pulp are cut off at the level of the apical foramen of such recently traumatized teeth resulting in ischemic infarction [5]. The epithelial lining is derived from the epithelial cell rests of Malassez and is thick, irregular, and continuous with squamous epithelium with granulation tissue forming the wall in denuded areas. Cholesterol crystal clefts and mucous cells may be found. The cyst fluid is usually watery but may be thick and syrupy, straw-coloured cholesterol crystal clefts. The straw-coloured aspirated liquid from the radicular cyst is usually shiny when placed on a cotton gauze and immediately viewed under bright light for a few seconds. This is known as the shimmers test. The radicular cyst usually has a fibrous connective tissue capsule [6]. Recent evidence suggests that radicular cyst formation is mediated by the presence of immunocompetent cells in the proliferating epithelium of periradicular lesion, eliciting an immunological reaction, which was confirmed by the presence of immunoglobulins in the cystic fluid. The epithelial cell rests on Malassez, gets antigenically recognized as antigens, and elicits the immunological reactions, resulting in the formation of a radicular cyst. The microbial products act as irritants, enter the inflamed periradicular tissues through the apical foramen of the affected teeth, and usually disintegrate spontaneously following the elimination of those microbial products by the presence of antigenic epithelium [7]. Bernardi et al. suggested that epithelial-stromal interaction is responsible for the sustenance and growth of radicular cysts [8].

Chen et al. histopathologically analyzed 232 cases of radicular cyst retrospectively and stated that 179 cases (77.2%) revealed chronic inflammatory infiltrate, 94 cases (40.5%) revealed dystrophic calcifications, 72 cases revealed foamy histiocytes (31.0%), 57 cases revealed haemosiderin (24.6%), 54 cases revealed cholesterol clefts (23.3%), 44 cases revealed foreign bodies (19.0%), 22 cases revealed bacterial colonies (9.5%), one case revealed Odontogenic epithelial rest (0.4%) [9]. Radicular cyst, if left untreated, can cause buccolingual expansion of the underlying bone resulting in facial asymmetry. In the maxilla and mandible's tooth-bearing areas, radicular cysts are common. Smaller lesions, which are inadvertently found during a radiological evaluation, have no symptoms. Large lesions are uncomfortable and painful due to subsequent infection. When the affected area of the cyst is palpated, it has a hard, bony consistency if the bone is still intact or a fluctuant consistency if the radicular cyst has destroyed the cortical tissue. Early detection of these cysts helps to prevent the pathological fracture that might occur as a result of the developing cysts, which cause thinning and resorption of bone. Such radicular cysts also can lead to pathological migration of the involved tooth, thinning followed by the destruction of the cortical plate caused by pressure effects of the enlarging cyst [10].

Kadam et al. recommended that the treatment of radicular cysts includes conventional nonsurgical root canal therapy when the lesion is localized or surgical treatment like enucleation, and marsupialization (Partsch operation), which involves the incomplete removal of the epithelial lining of the cyst or decompression when the lesion is large. The platelet-rich fibrin clot is placed for faster healing in the periapical region [11]. Larger radicular cysts associated with pathological bone destruction are managed by iliac bone graft [12]. The management modalities of Radicular cyst are described (Table 1).

**Table 1:** Management modalities of radicular cyst

Small-sized Radicular cyst (< 2 cm )	Surgical enucleation of the cyst/periapical curettage
Large-sized Radicular cyst (>2 cm)	Curettage or Marsupilization (Partsch operation), in which deroofing of the cystic wall is performed to relieve the pressure effects caused by the cyst
Large-sized Radicular cyst (>2cm) with a large underlying bone defect	Curettage followed by platelet -rich fibrin for faster healing . Bone grafts – autograft such as iliac bone graft / allograft

## Conclusions

The radicular cyst is the most common inflammatory odontogenic cyst in human jaws. Patients affected by such infected radicular cysts usually complain of pain and report to the oral physician. A careful history and clinical and radiological evaluation are necessary for patients to decide on treatment planning and efficient management of radicular cyst. In addition to being caused by trauma, radicular cysts can also grow as a result of injury to the periapical tissues beneath the tooth, such as from improper follow-up after a root canal procedure and from an access opening done on the root canal attempted tooth. When the patient has not followed up on further subsequent treatment visits, untreated radicular cysts may result in buccolingual growth of the underlying bone and asymmetric facial features. Radicular cysts are frequent in the tooth-bearing regions of the mandible and maxilla. Lesions accidentally discovered to be smaller during a radiological evaluation do not show any symptoms. Large lesions cause discomfort and agony because they subsequently become infected. When the damaged area of the cyst is palpated, it has a variable consistency such as firmness, when the cortical plate is intact or fluctuant, and if the radicular cyst has destroyed the cortical bone. Such infected radicular cyst can be better radiologically evaluated three-dimensionally with the help of CBCT than conventional intraoral periapical radiographs, which do not reveal the exact side of perforation of the cortical plate caused by expansion of the cyst. A CBCT is essential for the three-dimensional visualization of the radicular cyst. CBCT has an added advantage of high spatial resolution that helps to delineate the side of cortical plate destruction caused by radicular cysts and aid in definitive treatment planning.
